# A four-oscillator model of seasonally adapted morning and evening activities in *Drosophila melanogaster*

**DOI:** 10.1007/s00359-023-01639-5

**Published:** 2023-05-23

**Authors:** Taishi Yoshii, Aika Saito, Tatsuya Yokosako

**Affiliations:** Graduate School of Natural Science and Technology, Okayama University, Tsushima-Naka 3-1, Kita-ku, Okayama, 700-8530 Japan

**Keywords:** *Drosophila*, Seasonal adaptation, Photoperiod, Oscillator, Activity rhythm

## Abstract

The fruit fly *Drosophila melanogaster* exhibits two activity peaks, one in the morning and another in the evening. Because the two peaks change phase depending on the photoperiod they are exposed to, they are convenient for studying responses of the circadian clock to seasonal changes. To explain the phase determination of the two peaks, *Drosophila* researchers have employed the two-oscillator model, in which two oscillators control the two peaks. The two oscillators reside in different subsets of neurons in the brain, which express clock genes, the so-called clock neurons. However, the mechanism underlying the activity of the two peaks is complex and requires a new model for mechanistic exploration. Here, we hypothesize a four-oscillator model that controls the bimodal rhythms. The four oscillators that reside in different clock neurons regulate activity in the morning and evening and sleep during the midday and at night. In this way, bimodal rhythms are formed by interactions among the four oscillators (two activity and two sleep oscillators), which may judiciously explain the flexible waveform of activity rhythms under different photoperiod conditions. Although still hypothetical, this model would provide a new perspective on the seasonal adaptation of the two activity peaks.

## Introduction

Insects are small ectothermic animals that are vulnerable to harsh environments, such as hot and cold temperatures and desiccation, which change on a yearly cycle (Koštál 2011). To cope with these changes, insects have evolved the ability to adapt to seasonal changes in the environment, allowing them to live in a wide range of habitats, from the Arctic to the Antarctic. Therefore, understanding how insects adapt to seasonal changes is essential for understanding ecosystems.

Most insects exhibit daily rhythms, such as sleep–wake cycles and nocturnal/diurnal activity, in their behavior. Appropriate behavior at the right time of the day increases the efficiency of daily life and improves fitness (Vaze and Sharma 2013; Abhilash and Sharma 2016; Horn et al. 2019). Circadian behavioral rhythms (circa = about and dies = day from Latin terms) are controlled by a circadian clock that generates approximately 24 h rhythms in a self-sustaining manner. In addition, the clock uses environmental time cues, such as light and temperature, to reset the clock. In particular, sunlight is a reliable time cue as sunrise and sunset are indicative of the morning and evening, respectively.

**Fig. 1:**
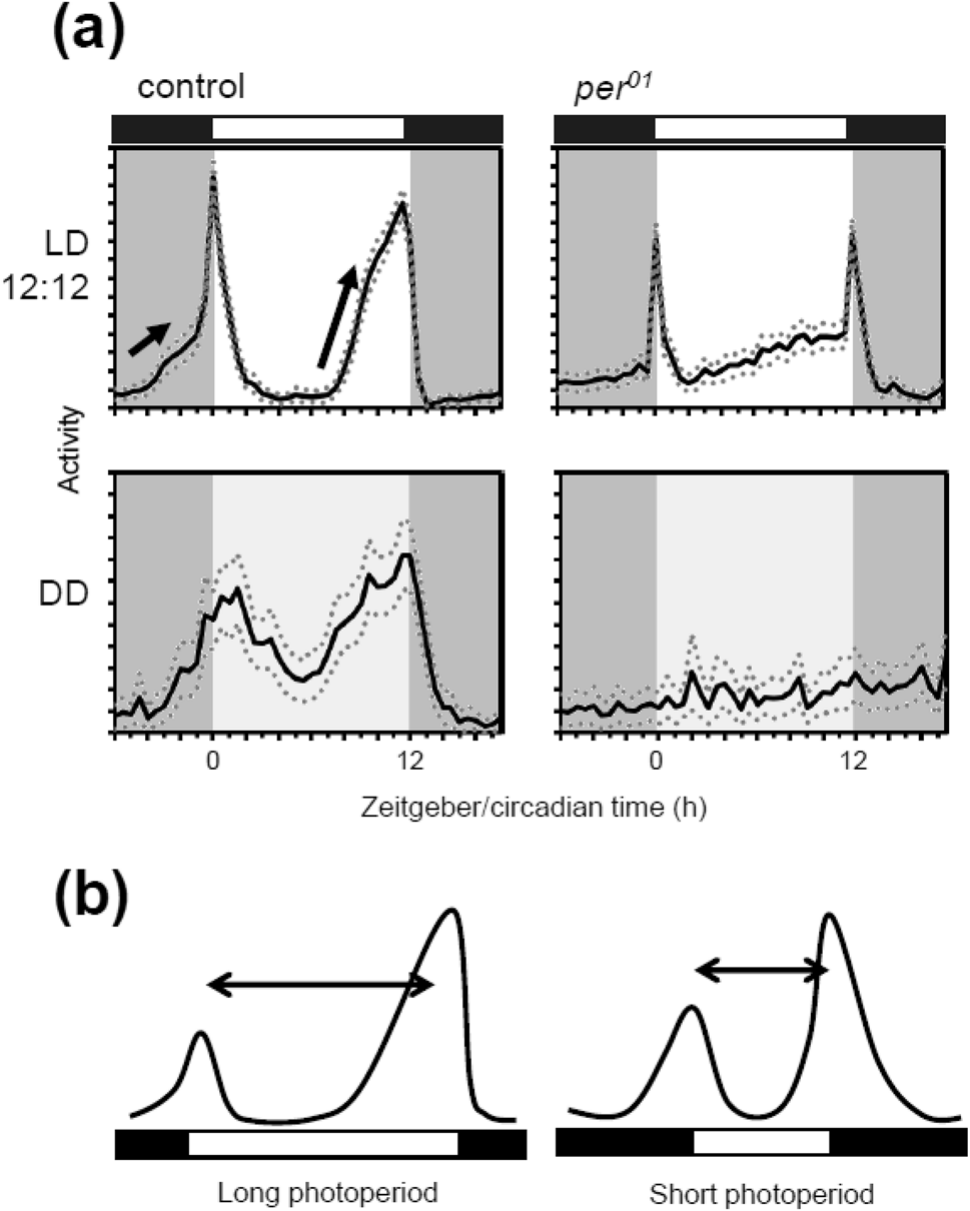
**a** Daily activity rhythms (mean ± standard error) of commonly used control (*w*^*1118*^, n = 30) and clock-less mutant (*per*^*01*^, n = 30) flies in LD12:12 and on the first day of DD after transfer from LD. Control flies show two activity peaks in the morning and evening, and each peak begins to increase before light transitions, known as anticipatory activity (arrows) in LD. The two peaks persist even in DD. *per*^*01*^ mutant flies also show two peaks; however, they are mere responses to light changes, known as the masking effects of light. Therefore, the two peaks in *per*^*01*^ flies do not persist in DD. **b** Phase relationship between the morning and evening peaks under long (left) and short (right) photoperiod conditions. The two peaks change phase with the given photoperiod. Under long photoperiod, the phase relationship between the two peaks is extended, whereas it is shortened under short photoperiod. In this way, flies change their active time depending on the season. The black and white boxes in the bars indicate dark and light conditions, respectively

The fruit fly, *Drosophila melanogaster,* is a small insect of 2–3 mm in size and is often used as a model organism in genetics (Hales et al. 2015). *Drosophila* is of the Afrotropical origin and has migrated to most continents along with humans (David and Capy 1988). They exhibit two activity peaks in the morning (M) and evening (E) under standard light–dark cycles of 12 h/12 h (LD12:12) (Fig. 1a) in the laboratory (Hamblen-Coyle et al. 1992; Helfrich-Förster 2000). The two peaks can persist and free-ran with a period of approximately 24 h under constant dark conditions (DD) (Fig. 1a) (Helfrich-Förster 2000), suggesting that the two peaks are generated by the circadian clock. Studies in *Drosophila* have significantly contributed to elucidate the molecular mechanisms of the circadian clock (King and Sehgal 2020; Beer and Helfrich-Förster 2020). Mechanisms underlying the circadian clock involve transcriptional/translational feedback loops composed of clock genes such as *per*, *timeless* (*tim*), *Clock*, and *cycle*.

**Fig. 2:**
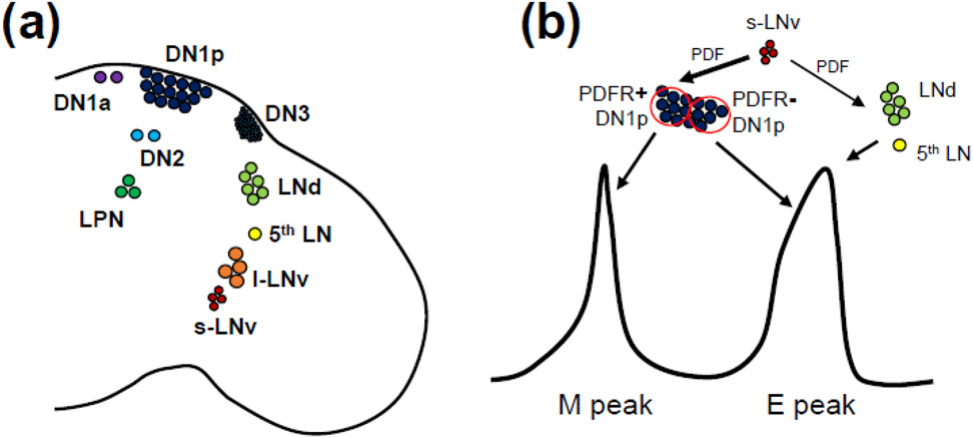
**a** Distribution of the clock neurons in the brain hemisphere of *Drosophila*. The circadian neuropeptide PDF is expressed in s-LNv and l-LNv groups. **b** Current model explaining how clock neurons control the M and E peaks. s-LNv neurons send PDF signals to PDF receptor (PDFR)^+^ DN1p neurons to control the M peak. s-LNv neurons send PDF signal to 5th LN and LNd neurons (PDFR^+^) also, which controls the E peak. PDFR^−^ DN1p neurons independently control the E peak

In *Drosophila*, the clock proteins PER and TIM are expressed in approximately 150 neurons in the brain (Fig. 2a) (Kaneko and Hall 2000). They are divided into the following clusters: small lateral neurons (s-LNvs), large lateral neurons (l-LNvs), 5^th^ lateral neuron (5^th^ LN, also known as 5^th^ s-LNv), dorsal lateral neurons (LNds), lateral posterior neurons (LPNs), anterior dorsal neurons 1 (DN1as), posterior dorsal neurons 1 (DN1ps), dorsal neurons 2 (DN2s), and dorsal neurons 3 (DN3s) (Ahmad et al. 2021; Crespo-Flores and Barber 2022). The s-LNv and l-LNv groups express a neuropeptide, Pigment-dispersing factor (PDF) (Helfrich-Förster 1995). Mutant flies lacking PDF show a tiny M activity peak, a phase-advanced E activity in LD, and fragile free-running rhythms in DD (Renn et al. 1999). Studies on the role of PDF in activity rhythms have shed light on how the clock is integrated into the neural network of the brain.

## Seasonal adaptation of M and E activity peaks

Under LD12:12 at 25 ℃, the standard experimental condition, the two activity peaks coincide when light is turned on and off (Fig. 1a). Both activity peaks begin to form before the light change, showing anticipatory activity to light. The two peaks can flexibly change their phase under different photoperiod conditions (Fig. 1b) (Rieger et al. 2003, 2012). Under short photoperiod conditions, the M and E peaks track light on and off, respectively. Therefore, the time interval between the M and E peaks (the phase angle between M and E peaks) becomes shorter than that under LD12:12 (Fig. 1b). In contrast, under long photoperiod conditions, the phase angle between M and E peaks becomes relatively long. The photoperiodic change of bimodal rhythms can also be observed in other animals and encodes seasonal information (Aschoff 1966; Pittendrigh and Daan 1976; Inagaki et al. 2007). The reason for phase changes of the two peaks in flies is explained as follows: By keeping the phase angle close between the two activity peaks, flies increase their activity during daytime in winter (short photoperiod conditions) to be active during warm periods. In contrast, by increasing the phase angle, flies reduce their activity during hot days in summer (long photoperiod conditions) to escape from heat and desiccation (Majercak et al. 1999), although this may apply to flies living in warm areas because northern flies respond differently to summer-like conditions (Kauranen et al. 2012; Menegazzi et al. 2017). The phase angle between the M and E peaks is not completely free and limits to expanding and contracting exist. These facts suggest that two different oscillators control the M and E peaks in the circadian clock as a single oscillator mechanism may not simultaneously change the phases of two peaks in opposite directions. The two oscillators track dawn and dusk independently; however, they are interlocked at some point to keep the phase angle. Given this two-oscillator model, one can imagine that the two oscillators are located in different groups of neurons that communicate with each other via neurotransmitters such as PDF (Dubruille and Emery 2008; Helfrich-Förster 2009; Yoshii et al. 2012).

Photoperiod gradually changes throughout the year and is a reliable indicator of the coming season. Although less reliable, ambient temperature is also a cue for the season (Majercak et al. 1999; Bywalez et al. 2012). Under low-temperature or short-day conditions with temperature cycles, the M peak is phase-delayed, and the E peak is phase-advanced to increase diurnal activity, which is similar to the M and E peaks under short photoperiod conditions. Under high-temperature or long-day conditions with temperature cycles, the two peaks show opposite responses, similar to those under long photoperiod conditions. Therefore, the two oscillators controlling the M and E peaks can respond similarly to photoperiod and ambient temperature. Notably, the effects of photoperiod and temperature on the two peaks are not exactly the same. Activity of the M peak is increased and decreased in short and long photoperiod conditions, respectively (Rieger et al. 2003). Conversely, it is decreased and increased in relatively cool and warm conditions, respectively (Bywalez et al. 2012). Therefore, the M and E oscillators can have different outputs to regulate the activity of M in response to photoperiod and temperature.

## Morning and evening oscillators based on clock neurons

Different clusters of clock neurons differ in neurite morphology and neurotransmitter content, suggesting that they have distinct roles (Helfrich-Förster et al. 2007). The discovery of PDF has facilitated functional analysis of PDF-positive clock neurons, the s-LNv and l-LNv groups. The tiny M peak of *Pdf* mutants in LD suggests that PDF-positive clock neurons play a role in generating the M peak (Renn et al. 1999; Stoleru et al. 2004). *per*-rescue experiments in PDF neurons only have showed the restoration of the anticipatory M peak but not of the E peak (Grima et al. 2004). In contrast, *per*-rescue and cell-ablation experiments in 5th LN and LNd neurons have shown that these clock neurons are essential for the E peak (Grima et al. 2004; Stoleru et al. 2004). The underlying reason for phase advancement of the E peak in *Pdf* mutants compared to that in control flies can be explained by the interaction of PDF neurons (M oscillators or M neurons) with 5th LN and LNd neurons (E oscillators or E neurons) via PDF (Peng et al. 2003; Stoleru et al. 2005; Shafer et al. 2008; Yoshii et al. 2009). Intracellular Ca^2+^ concentration in clock neurons oscillates in a circadian manner (Liang et al. 2016). The Ca^2+^ levels of M and E neurons peak in the morning and evening, respectively. In *Pdf*^*01*^ mutant flies, the Ca^2+^ level in E neurons is phase-advanced according to their activity rhythms, whereas that in M neurons is not affected (Liang et al. 2017). Therefore, M and E neurons inherently have two different phases, and PDF signaling from M to E neurons modulates the phase of E neurons.

The period of circadian molecular oscillation can be genetically accelerated or decelerated by overexpressing mutated Doubletime or Shaggy kinases (Stoleru et al. 2005). Such genetic manipulations in M neurons have revealed that the molecular clock in two LNd neurons [PDF receptor (PDFR)-positive] is dictated by M neurons, and other PDFR-positive LNd and 5th LN neurons are somewhat influenced by M neurons, whereas PDFR-negative three LNd neurons are completely insensitive to M neurons (Yao and Shafer 2014). Therefore, E neurons contain three populations with different coupling strengths to M neurons. This heterogeneity of E neurons may simultaneously explain the flexibility and rigidity of the phase angle between the M and E peaks. The direction of coupling between M and E neurons changes depending on the photoperiod (Stoleru et al. 2007). M neurons dominate E neurons under short photoperiod conditions, and the opposite phenomenon takes place under long photoperiod conditions. The coupling pathway from M to E neurons is mediated by PDF; however, the pathway in the opposite direction remains unknown.

The M–E oscillator model mentioned above does not exclude the involvement of other clock neurons in generating the M and E peaks. Depending on temperature and illumination, DN1p neurons alone are sufficient for the M and E peaks (Zhang et al. 2010). DN1p neurons consist of approximately 15 neurons per hemisphere. Half of them express PDFR, a glutamate transporter, diuretic hormone 31, and cryptochrome (CRY), a protein involved in light entrainment (Yoshii et al. 2008; Im and Taghert 2010; Kunst et al. 2014; Guo et al. 2016; Goda et al. 2016; Chatterjee et al. 2018). The two types of DN1p neurons have different projection patterns (Chatterjee et al. 2018; Lamaze et al. 2018; Guo et al. 2018; Reinhard et al. 2022b). Chatterjee et al. (2018) has proposed that the PDFR-positive DN1p group is responsible for the M peak, and the PDFR-negative DN1p group is responsible for the E peak. s-LNv neurons (M oscillators) interact with the PDFR-positive DN1p group via PDF to generate the M peak, whereas three different E oscillator neurons, the 5^th^ LN, LNd, and PDFR-negative DN1p groups, can control the E peak relatively independently (Fig. 2b).

Menegazzi et al. (2020) have conducted *per*-rescue experiments under long and short photoperiod conditions. The original M and E oscillator model of seasonal adaptation is based on the two oscillators flexibly tracking dawn and dusk under different photoperiods (Pittendrigh and Daan 1976). They found that flies with *per*-rescue in all clock neurons showed phase adjustment of the M and E peaks similar to that of the wild-type flies under different photoperiods. However, flies with *per*-rescue only in M or E neurons did not show this phenomenon. Therefore, although M and E neurons are essential for generating the M and E peaks, some unidentified key mechanism regulates normal phase adjustment of the two peaks under long and short photoperiod conditions. The possibilities include: 1. Other clock neurons that are not classified as M or E neurons and play a role in the two activity peaks and 2. The whole neural network between M and E neurons, which is important for the phase adjustment of the two peaks. For the first hypothesis, for example, the roles of DN1a, DN3, and LPN groups in activity rhythms and sleep have been demonstrated (Fujiwara et al. 2018; Reinhard et al. 2022a; Sun et al. 2022). These groups of clock neurons may cooperate with M and E neurons. For the second hypothesis, disruption of the clock neuronal network reduces the strength of free-running rhythms in DD (Bulthuis et al. 2019; Jaumouillé et al. 2021). This suggests that manipulating specific groups of clock neurons may disrupt proper network interactions, causing the entire network to collapse and produce aberrant M and E activity rhythms. Of course, a third hypothesis that is a blend of these two hypotheses must also be considered.

## Molecular oscillations under different photoperiods

The phases of molecular oscillations do not differ between M and E neurons. For example, PER and TIM levels peak late at night in all groups of clock neurons. Therefore, molecular oscillations generate circadian rhythms, and output pathways determine the phases of two activity peaks (Liang et al. 2016). However, molecular oscillations also play a role in their phase determination. An exposure to dim light at night causes a phase-advance of the M peak and phase-delay of the E peak (Bachleitner et al. 2007; Kempinger et al. 2009). In this situation, the phases of PER and TIM peaks are phase-advanced in M neurons and phase-delayed in E neurons, consistent with the phase changes of M and E peaks (Bachleitner et al. 2007). Therefore, the phases of the two peaks would be determined first in the molecular oscillations and then in the downstream pathways.

The first attempt to monitor PER and TIM oscillations in clock neurons under long and short photoperiods was made by Shafer et al. (2004). They found that the phases of nuclear accumulation of PER and TIM coincided under a short photoperiod, whereas, under a long photoperiod, nuclear accumulation of PER peaked when the level of TIM was very low after exposure to light. A similar decoupling of PER and TIM cycling was observed in flies living under long photoperiods in natural conditions (Menegazzi et al. 2013). Kistenpfennig et al. (2018) immunostained PAR domain protein 1 (PDP1) in LD12:12 and LD20:4 and noticed that the phases of PDP1 cycling between M and E neurons were almost the same in the two photoperiods, but the amplitude of PDP1 cycling in all clock neurons was high in LD12:12 and low in LD20:4. Therefore, the phases of molecular oscillations and their amplitude may encode phase determination of the M and E peaks. The amplitude of PDP1 cycling may be the result of the coupling strength of PER and TIM cycling. When PER and TIM rhythms are decoupled under long photoperiod conditions, the entire circadian feedback loops may be degraded owing to the prolonged but weak activities of PER and TIM, resulting in the low amplitude of PDP1 cycling.

The present model based on clock neurons is already complicated, and molecular oscillations under different photoperiods add further complexity. Applying the classical two-oscillator model to what is being observed in present molecular, neural, and behavioral data is being increasingly difficult. Therefore, new concepts to explain the phase adjustment of M and E activities should be considered.

## Should more oscillators be added?

We spontaneously think that the oscillators generate activity peaks. This is true because *per*^*01*^ mutants do not show the M and E peaks with anticipation of dawn and dusk, except for masking effects to light (Fig. 1a). In addition, *per*^*01*^ mutants do not show siesta and night sleep. Therefore, assuming a negative oscillator to suppress activity (or to promote sleep) may be appropriate.

**Fig. 3:**
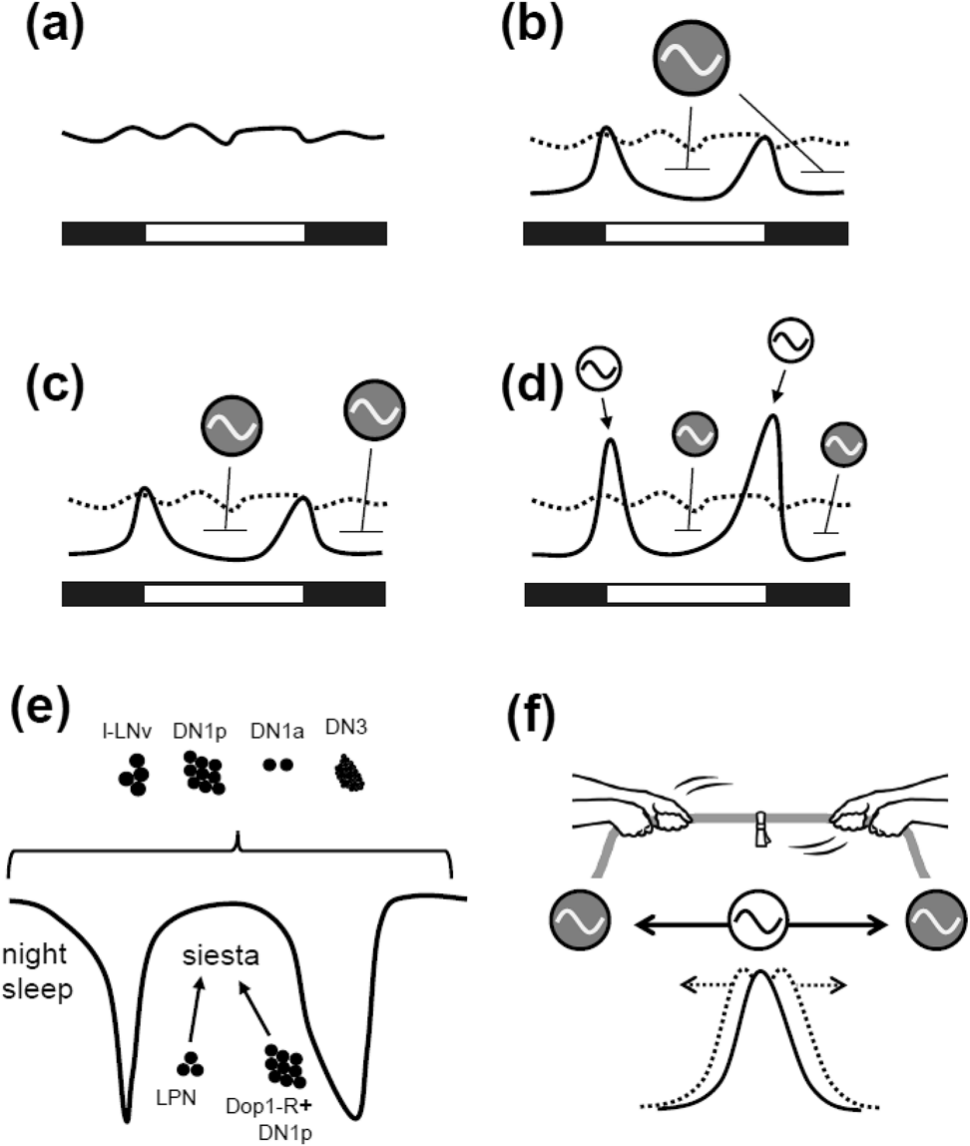
Oscillator models to explain the occurrence of M and E peaks. **a** Daily activity without masking effects of light in a clockless condition. The two activity peaks are absent. **b** One sleep oscillator suppresses daytime and nighttime activities, resulting in two peaks in the morning and evening. **c** Two sleep oscillators (siesta and night sleep) produce two peaks. **d** Additional M and E oscillators promote two activities in the morning and evening, generating two distinct peaks. **e** l-LNv, DN1a, DN1p, LPN, and DN3 clock neurons influence sleep. In particular, Dop1-R positive DN1p and LPN neurons are responsible for regulating siesta. **f** The active oscillator (M or E oscillator) interacts with the sleep oscillators on both sides to determine the phase of activity peak

For example, a sleep oscillator outputs two signals during the day and night, resulting in two activity peaks in the morning and evening (Fig. 3b). This is enough to produce the two peaks. We then split the sleep oscillator into two sleep oscillators for siesta and night sleep (Fig. 3c). If the two sleep oscillators respond to photoperiods by changing sleep duration, the M and E peaks can be phase-shifted as if they are tracking dawn and dusk, respectively. Importantly, the siesta oscillator should work in conjunction with the night sleep oscillator to provide a flexible phase angle between the M and E peaks.

Sleep is regulated by some groups of clock neurons. The l-LNv group has been first proposed as the clock neurons responsible for sleep regulation (Shang et al. 2008, 2011; Sheeba et al. 2008; Parisky et al. 2008; Chung et al. 2009; Lebestky et al. 2009; Gmeiner et al. 2013). Later, sleep studies were expanded into other clock neuron groups such as DN1a, DN1p, LPN, and DN3 (Kunst et al. 2014; Guo et al. 2016, 2018; Fujiwara et al. 2018; Lamaze et al. 2018; Ni et al. 2019; Reinhard et al. 2022a; Sun et al. 2022; Schlichting et al. 2022). Schlichting et al. (2022) showed that a subset of DN1p neurons expressing two dopamine receptors, Dop1R1 and Dop1R2, is particularly important for regulating siesta (Fig. 3e). In addition, LPN neurons affect siesta (Fig. 3e; Ni et al. 2019; Reinhard et al. 2022a). Therefore, a specific group of clock neurons may be responsible for night sleep. The peak phases of Ca^2+^ rhythms in s-LNv and LNd neurons correspond to the M and E peaks, respectively, while those in l-LNv and DN1p neurons correspond to midday and midnight (Liang et al. 2016, 2017, 2019). If the trough of Ca^2+^ rhythms in DN1p neurons affects siesta, that in l-LNv neurons may be responsible for night sleep. Since activity and sleep patterns are closely related, the neurons that control activity and sleep would interact with each other. Some DN and LPN neurons play a role in temperature entrainment (Yoshii et al. 2005, 2010; Miyasako et al. 2007; Chen et al. 2015, 2018; Harper et al. 2016; Reinhard et al. 2022a). Their temperature sensitivity may contribute to integrating seasonal temperature inputs into sleep regulation.

After assuming the two sleep oscillators, the M and E activity oscillators are added to the model (Fig. 3d). The sleep oscillators suppress activity (or promote sleep), while the M and E oscillators increase activity in the morning and evening. As the inhibitory effect of the night sleep oscillator diminishes, and the M oscillator becomes active at dawn, M activity gradually increases before exposure to light in the morning, creating anticipatory activity. Therefore, the anticipatory M activity can be explained by the inferiority and superiority of the night sleep oscillator relative to the M oscillator. The same can be imagined for controlling the phase of E activity by an interaction between the siesta and E oscillators. Phase determination of the two peaks can also be explained by a tug-of-war between the two sleep oscillators, with the active oscillator in the middle as a rope (Fig. 3f). The active oscillator has its own phase, but the two sleep oscillators modulate it. The dominance of oscillators can change with day length, and their tug-of-war determines the seasonally adapted activity phases.

The model consisting of four oscillators, including the M, siesta, E, and night sleep, would not be a perfect option to explain all phenomena observed in the rhythms of *Drosophila* activity. However, it has the advantage that sleep regulation can be taken into account in phase determination of the two activity peaks.

## Concluding remarks

The reason for conceiving the sleep oscillators is that we often face the difficulty of quantifying anticipatory activity for the M and E peaks. In *Drosophila*, anticipatory activity is measured by the activity of flies prior to changes in light at dawn and dusk (Harrisingh et al. 2007). However, a large variation is noticed in anticipatory activity even in the control strains. We noticed that activity before dawn and dusk and night sleep and siesta contributed to the distinct anticipatory activity. Liang et al. (Liang et al. 2016, 2017, 2019, 2023) proposed that different groups of clock neurons generate intracellular Ca^2+^ rhythms in different phases, recalling the possibility of a multioscillator system (Yoshii et al. 2004; Rieger et al. 2006; Miyasako et al. 2007). The four-oscillator model proposed here is an idea only. Sometimes, however, assuming a simple model can lead to a better understanding of complex phenomena, as was the case with the two-oscillator model of Pittendrigh and Daan (1976).

## Data Availability

This article has no additional data.
